# Pigs Overexpressing Porcine β-Defensin 2 Display Increased Resilience to *Glaesserella parasuis* Infection

**DOI:** 10.3390/antibiotics9120903

**Published:** 2020-12-14

**Authors:** Jing Huang, Xiaoyu Yang, Antian Wang, Chao Huang, Hao Tang, Qiuhong Zhang, Qiong Fang, Zuming Yu, Xiao Liu, Qi Huang, Rui Zhou, Lu Li

**Affiliations:** 1 State Key Laboratory of Agricultural Microbiology, College of Veterinary Medicine, Huazhong Agricultural University, Wuhan 430070, China; jing_huang@webmail.hzau.edu.cn (J.H.); yangxiaoyu0118@webmail.hzau.edu.cn (X.Y.); wangantian@webmail.hzau.edu.cn (A.W.); huangchao_1991@webmail.hzau.edu.cn (C.H.); 2019302110155@webmail.hzau.edu.cn (H.T.); zhangqiuhong@webmail.hzau.edu.cn (Q.Z.); fangqiong@webmail.hzau.edu.cn (Q.F.); yzm@webmail.hzau.edu.cn (Z.Y.); liuxiao324@webmail.hzau.edu.cn (X.L.); qhuang@mail.hzau.edu.cn (Q.H.); 2 Cooperative Innovation Center for Sustainable Pig Production, College of Veterinary Medicine, Huazhong Agricultural University, Wuhan 430070, China; 3 International Research Center for Animal Disease, Ministry of Science and Technology of China, Wuhan 430070, China; 4 Key Laboratory of Development of Veterinary Diagnostic Products, Ministry of Agriculture and Rural Affairs of China, Wuhan 430070, China

**Keywords:** porcine β-defensin 2, transgenic pigs, *Glaesserella (Haemophilus) parasuis*, disease-resistant animal, antimicrobial peptide, antibacterial activity

## Abstract

As the causative agent of Glässer’s disease, *Glaesserella (Haemophilus) parasuis* has led to serious economic losses to the swine industry worldwide. Due to the low cross-protection of vaccines and increasing antimicrobial resistance of *G. parasuis*, it is important to develop alternative approaches to prevent *G. parasuis* infection. Defensins are host defense peptides that have been suggested to be promising substitutes for antibiotics in animal production, while porcine β-defensin 2 (PBD-2) is a potent antimicrobial peptide discovered in pigs. Our previous study generated transgenic (TG) pigs overexpressing PBD-2, which displayed enhanced resistance to *Actinobacillus pleuropneumoniae*. In this study, the antibacterial activities of PBD-2 against *G. parasuis* are determined in vitro and in the TG pig model. The concentration-dependent bactericidal activity of synthetic PBD-2 against *G. parasuis* was measured by bacterial counting. Moreover, after being infected with *G. parasuis* via a cohabitation challenge model, TG pigs overexpressing PBD-2 displayed significantly milder clinical signs and less severe gross pathological changes than their wild-type (WT) littermates. The TG pigs also exhibited alleviated lung and brain lesions, while bacterial loads in the lung and brain tissues of the TG pigs were significantly lower than those of the WT pigs. Additionally, lung and brain homogenates from TG pigs possessed enhanced antibacterial activity against *G. parasuis* when compared with those from the WT pigs. Altogether, these proved that overexpression of PBD-2 could also endow pigs with increased resilience to *G. parasuis* infection, which further confirmed the potential of using the PBD-2 coding gene to develop disease-resistant pigs and provided a novel strategy to combat *G. parasuis* as well.

## 1. Introduction

Defensins are endogenous antimicrobial peptides that are widely distributed in vertebrates, invertebrates, plants, and fungi [1,2]. They constitute a major family of host defense peptides, which have been suggested to be attractive alternatives to the usage of antibiotics in animal production [3,4]. Mammalian defensins are classified as α-, β- or θ-defensins in accordance to their structural characteristics [5]; they possess multiple biological functions, including mediating resistance to microorganisms as well as immune regulation [6,7]. Genes encoding defensins have been widely used as candidates to produce disease-resistant animals. For example, overexpression of the mouse defensin Bin1b in mice could protect against *Escherichia coli* infection [8]. Transgenic expression of human defensin 5 in mice conferred enhanced resistance to *Salmonella enterica* serotype Typhimurium infection [9]. Moreover, alveolar epithelial cells and macrophages from transgenic (TG) cattle expressing human β-defensin 3 were less susceptible to *Mycobacterium bovis* infection [10].

Within the porcine defensin family, only β-defensins have been discovered [11]. Amongst 27 β-defensins which have been found in pigs so far [12], porcine β-defensin 2 (PBD-2) has been shown to exert significant antibacterial properties against a broad range of both Gram-positive and Gram-negative bacteria [6,13]. Recombinant PBD-2 has been successfully used as a feed additive for weaned piglets to reduce the incidence of postweaning diarrhea and to improve growth performance of piglets [14]. The combined use of PBD-2 as a feed additive not only promoted the growth of juvenile goats but also improved their rumen microbial community structure [15]. Another study revealed that PBD-2 could provide protection against *S.* Typhimurium infection in TG mice through direct bactericidal ability and the inactivation of the TLR4/NF-κB pathway [16]. In addition, mice expressing PBD-2 became more resistant to pseudorabies virus (PRV) infection [17], while pigs overexpressing PBD-2 obtained improved resilience to *Actinobacillus pleuropneumoniae* infection [18]. Additionally, fibroblasts from our recently produced PBD-2 TG pigs displayed enhanced resistance to both *Streptococcus suis* and *A. pleuropneumoniae* infections [19].

*Glaesserella (Haemophilus) parasuis*, as the etiologic agent of Glässer’s disease, colonizes the upper respiratory tract of swine and is responsible for large economic losses to the global swine industry [20]. *G. parasuis* infection in pigs mainly manifests as fibrinous polyserositis, arthritis, and meningitis [21]. As a primary pathogen of pigs, *G. parasuis* can coinfect with other pathogens including *S. suis*, porcine reproductive and respiratory syndrome virus (PRRSV), porcine circovirus, and *Bordetella bronchiseptica* [22,23,24,25]. The complexity of coinfections in herds has led to difficulties in the diagnosis and treatment of Glässer’s disease.

To date, 15 serovars of *G. parasuis*, with different degrees of virulence, have been identified [26]. The prevalent serovars of *G. parasuis* vary in different geographical regions. Serovars 5, 4, 13 and 2 are prevalent in some European countries [27,28]. The prevalent serovars in Brazil are 4, 5, 14, and 13 [29]. In China, serovars 4 and 5 are the most prevalent, while other serovars are also distributed nationwide [30,31]. Serovar 5 has been reported to be one of the most prevalent serovars in Central Vietnam, followed by serovars 2, 4, 10, and 9 [32]. Nowadays, commercially available *G. parasuis* vaccines are used to protect pigs against *G. parasuis* infection [33]. However, poor vaccine efficacy is frequently reported due to the lack of cross-protection against different serovars of *G. parasuis* [34]. Additionally, antimicrobial resistance among *G. parasuis* is of increasing global concern. For instance, 7 out of 60 *G. parasuis* isolates from Spain showed multiresistance to at least eight antimicrobials [35]; 110 *G. parasuis* isolates from five Southern China provinces exhibited high antimicrobial resistance to four antibiotics [36]. Additionally, within the 123 *G. parasuis* isolates from Germany, high minimal inhibitory concentration values for at least six antibiotics were detected [37]. Therefore, it is high time that we should come up with alternative approaches, such as breeding disease-resistant swine herds, to control *G. parasuis* infection.

To explore potential therapeutic and prophylactic methodologies against Glässer’s disease, this study aims to determine the antibacterial activity of PBD-2 against *G. parasuis* both in vitro and in TG pigs, which would also contribute to further use of the gene coding for PBD-2 as a potential candidate to generate disease-resistant animals.

## 2. Results

### 2.1. Antibacterial Activity of Synthetic PBD-2

**Figure 1:**
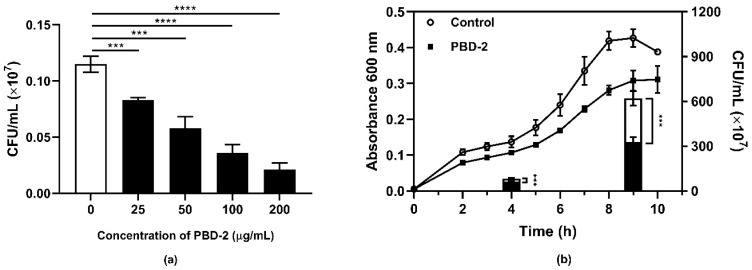
Inhibition of the growth of *Glaesserella parasuis* by synthetic porcine β-defensin 2 (PBD-2). (**a**) PBD-2 at various concentrations was incubated with *G. parasuis* for 1 h before plating and counting the surviving bacteria. Bacterial culture without PBD-2 treatment was used as a negative control. (**b**) The growth curves of *G. parasuis* cultured with and without PBD-2 (500 µg/mL) were drawn by recording values of optical density at 600 nm every hour until the stationary phase. The bacterial numbers in mid-log and stationary phases were determined by plate counting. The black-framed white column contains the black column below. Data are presented as mean ± SD and plotted from three independent experiments. *** *p* < 0.001, **** *p* < 0.0001, unpaired one-tailed Student′ s *t*-test.

To evaluate the bactericidal ability of synthetic mature PBD-2, *G. parasuis* SH0165 was incubated with or without PBD-2 of different concentrations. The bactericidal activity of PBD-2 was analyzed by converting the corresponding number of bacterial colonies on agar plates. As shown in Figure 1a, there was a significant reduction in the number of surviving bacteria compared with the control group. Additionally, the number of surviving bacteria decreased as the concentration of PBD-2 increased. These results indicate that synthetic PBD-2 has a dose-dependent direct bacterial killing effect against *G. parasuis*. The growth curves of *G. parasuis* cultured with or without PBD-2 additionally demonstrated that the growth of *G. parasuis* was significantly attenuated by PBD-2, while bacterial numbers of the group cultured with PBD-2 were significantly reduced in both the mid-log and stationary phases compared to the control group (Figure 1b). Likewise, similar bactericidal effects of PBD-2 on other 14 *G. parasuis* serovars were observed (Figure S1), which implies common bactericidal activities of PBD-2 on different *G. parasuis* strains.

### 2.2. Clinical Monitoring during Cohabitation

**Figure 2:**
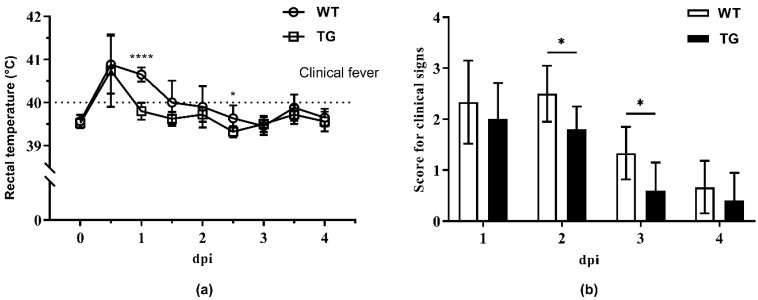
Clinical monitoring during cohabitation. Transgenic (TG, *n* = 5) and wild-type (WT, *n* = 6) pigs were cohoused with 11 other WT pigs intratracheally infected with *G. parasuis*. (**a**) Rectal temperature of pigs was recorded during the four-day cohabitation postinfection. (**b**) Scores for clinical signs in both WT and TG groups during the four-day cohabitation postinfection. Data are presented as mean ± SD. * *p* < 0.05, **** *p* < 0.0001, unpaired one-tailed Student′ s *t*-test.

To mimic the natural infection of Glässer’s disease in swine herds, an in-contact challenge trial was performed. Five TG pigs and six wild-type (WT) littermates were raised along with 11 WT pigs intratracheally challenged with *G. parasuis*. During cohabitation, body temperatures and clinical signs of those pigs without the intratracheal challenge of the *G. parasuis* SH0165 were recorded daily. As shown in Figure 2a, fever (>40 °C) occurred within one day postinfection and persisted longer in the WT group, while the rectal temperature of the TG group dropped back to a normal level (<40 °C) within 24 h. Though clinical signs of depression, uncoordinated movement, coughing, and sneezing were observed in both WT and TG groups (Table S1), the overall score for clinical signs in the TG group was significantly lower than that in the WT group (Figure 2b). In contrast, pigs in the mock group showed no clinical signs (Table S1).

### 2.3. Postmortem Analysis

**Figure 3:**
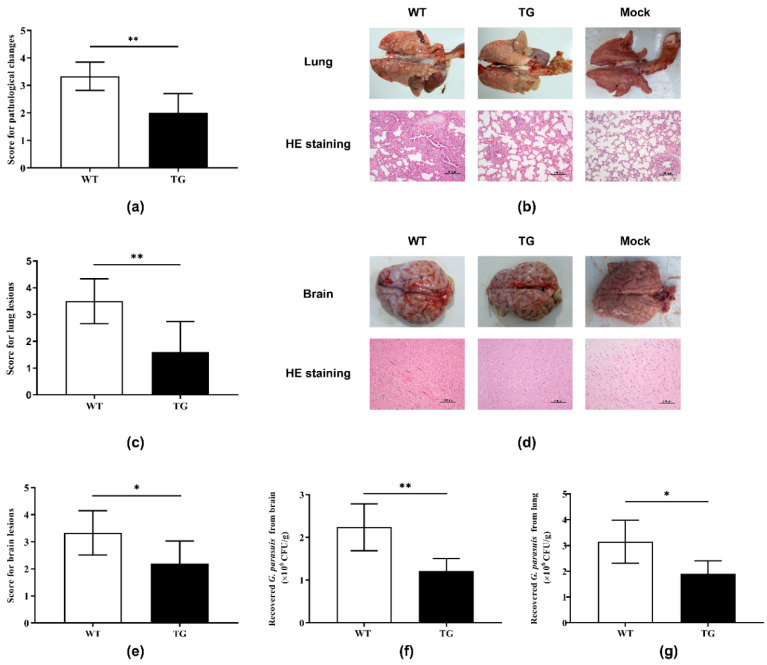
Pigs overexpressing PBD-2 exhibited reduced susceptibility to *G. parasuis* infection. (**a**) Assessment on the gross pathological changes of WT (*n* = 6) and TG (*n* = 5) pigs, with higher scores representing greater disease severity. (**b**) Representative macroscopic and microscopic changes of lungs. Mock: pigs without any bacterial infection. (**c**) Scoring for lung lesions caused by *G. parasuis* infection. (**d**) Representative macroscopic and microscopic changes of brains. (**e**) Scoring for brain lesions caused by *G. parasuis* infection. (**f**) Bacterial loads of *G. parasuis* in brain tissues of the pigs. (**g**) Bacterial loads of *G. parasuis* in lung tissues of the pigs. Data are represented by means ± SD. * *p* < 0.05; ** *p* < 0.01, unpaired one-tailed Student′ s *t*-test.

After the cohabitation described above, all pigs were sacrificed for subsequent postmortem analysis. In addition to meningitis, hepatitis, and pneumonia found in both TG and WT pigs, pericarditis was also detected in WT pigs, while pigs in the mock group did not show any pathological changes (Table S2). The score for pathological changes in the TG group was significantly lower than that in the WT group (Figure 3a), indicating relieved disease severity among TG pigs. Histopathological examination of lung tissue sections revealed lesions in both the WT and TG groups, which included alveolar wall thickening, inflammatory cell infiltration, and thrombi (Figure 3b). However, the extent of lung lesions in the TG pigs was milder than that of the WT pigs, shown as a significantly lower score for lung lesions in comparison to that of the WT pigs (Figure 3c). Hematoxylin and eosin (HE) staining of brain tissues showed that focal bleeding within white matter, widened perivascular spaces, and perivascular inflammatory infiltration were observed in WT pigs, whereas only mild perivascular inflammatory infiltration was found in TG pigs (Figure 3d). Consequently, the score for brain lesions of the TG pigs was significantly lower than that for the WT pigs (Figure 3e). Meanwhile, pigs in the mock group did not demonstrate any lesions in lungs or brains (Figure 3b,d).

Moreover, bacterial loads of *G. parasuis* in brains and lungs from both WT and TG pigs were quantified by counting bacterial colonies on agar plates. As demonstrated in Figure 3f, the TG pigs carried significantly fewer *G. parasuis* bacteria in their brains compared with their WT littermates. Similarly, *G. parasuis* bacterial loads in the lungs of TG pigs were significantly lower than those of WT pigs (Figure 3g). Taken together, these results indicate that TG pigs overexpressing PBD-2 acquire enhanced resistance to *G. parasuis* infection.

### 2.4. Bactericidal Activity of Tissue Homogenates

**Figure 4:**
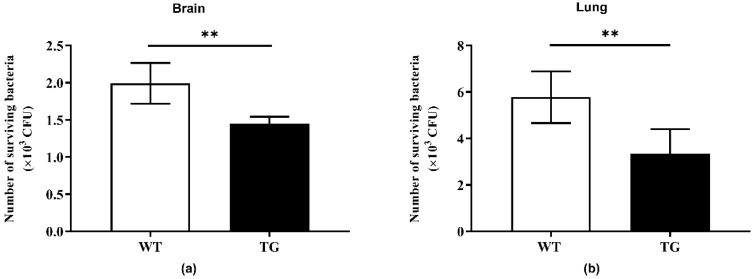
Brain (**a**) and lung (**b**) homogenates from the TG pigs (*n* = 5) displayed enhanced antibacterial activity against *G. parasuis* when compared with those from the WT pigs (*n* = 6). Brain or lung tissues of 400 mg from each pig were homogenized and centrifugated, and heat-inactivated supernatants were mixed with *G. parasuis* (2.5 × 10^3^ colony-forming units) at 37 °C for 1 h. The number of surviving bacteria in the mixture was counted. Data are presented as mean ± SD and plotted from three independent experiments. ** *p* < 0.01, unpaired one-tailed Student’ s *t*-test.

To investigate whether overexpression of PBD-2 could render pigs more resistant to *G. parasuis* infection, supernatants of brain and lung homogenates from both TG and WT pigs were collected. The supernatants were heat-inactivated before incubation with *G. parasuis*, followed by plate counting for the analysis of the bactericidal activity of the supernatants. The number of viable bacteria of the TG group was significantly lower than that of the WT group (Figure 4), suggesting that TG pigs were endowed with improved resistance against *G. parasuis* infection due to overexpressed PBD-2 in different tissues.

## 3. Discussion

As regards viable alternatives to antibiotics, defensins have been reported to have effective antimicrobial activities against infectious agents, including bacteria. Within the 29 porcine β-defensins discovered so far, the antibacterial activities of porcine β-defensin 1, PBD-2, and porcine β-defensin 129 have been well described [6,38,39]. Notably, since the discovery of PBD-2 in 2006 via sequence similarity analysis [40], PBD-2 has been the most studied porcine defensin regarding its antibacterial property. Synthetic PBD-2 has been proven to possess broad-spectrum antimicrobial abilities against bacterial pathogens [6]. In addition, the inhibition effect on the growth of *G. parasuis* by recombinant PBD-2 expressed by *Pichia pastoris* and *Bacillus subtilis* has been respectively characterized though the recombinant PBD-2 carries an extra N- or C-terminus [13,41]. The influence of these termini on the bioactivity of PBD-2, especially bactericidal activity against *G. parasuis*, remains unknown. Therefore, this study further confirmed the anti-*G. parasuis* activity of PBD-2 using synthetic mature PBD-2. Given that PBD-2 overexpression rendered pigs resistant to *A. pleuropneumoniae* infection [18], this study additionally proved that PBD-2 TG pigs exhibited increased resistance to another important pig pathogen (*G. parasuis*), which indicated that TG pigs overexpressing PBD-2 might be resistant to various infectious diseases.

*G. parasuis* is present in all swine-producing countries and results in large economic losses to the swine industry each year [20]. Commercial vaccines only provide protection against a specific group of serovars, instead of all serovars of *G. parasuis* [42]. The emerging antibiotics resistance in *G. parasuis* also brings about difficulties in controlling this pathogen [43]. In recent years, different attempts, other than using antibiotics, to prevent and control *G. parasuis* have been reported. He et al. claimed that *Blumea balsamifera* DC. essential oil possessed direct bactericidal activity against *G. parasuis* [44]. In addition, emodin from *Polygonum cuspidatum* inhibited *G. parasuis* essential metabolic pathways and bacterial cell division, thereby inhibiting the growth of *G. parasuis* [45,46]. Baicalin could alleviate *G. parasuis*-induced cell apoptosis by regulating the PKC-MAPK signaling pathway [47]. This study also provided the practical idea of breeding TG pigs overexpressing PBD-2 to combat *G. parasuis* infection in pig production. 

In terms of the antibacterial activities of defensins, previous studies have described both direct and indirect mechanisms of antibacterial action. Generally, defensins can cause bacterial membrane disruption, followed by cytolysis and leakage of intracellular compounds [48,49,50,51]. Additionally, defensins were able to block bacterial cell wall biosynthesis through binding to lipid II [52]. Human α-defensin 6 has been found to self-assemble into oligomers to agglutinate bacteria [53]. Additionally, tick defensin is capable of inhibiting cell division by inducing multiple cross-walls in bacteria [48]. The antibacterial activity of scorpion defensin BmKDfsin4 is associated with its ability to block potassium channels [54]. Moreover, human β-defensin 118 inhibits macromolecular synthesis in *E. coli* [49], while manila clam defensin Rpdef1α prevents biofilm formation of *E. coli* [50]. The ability of avian defensins to chemoattract immune cells also contribute to their antibacterial activities [51]. Although the antibacterial mechanism of PBD-2 against *Staphylococcus aureus* and *E. coli* has been characterized as being membrane disruption and regulation of DNA transcription and translation after PBD-2 is present in the cytoplasm [55,56], the antibacterial mechanism of PBD-2 against *G. parasuis* is unclear and requires further studies. 

The rapid development of transgenic technologies has made it possible to generate pigs with increased resilience to infectious diseases. For instance, pigs overexpressing histone deacetylase 6 acquired enhanced resistance to PRRSV [57]. Integration of specific antiviral small hairpin RNAs (shRNA) into the genome could protect pigs from PRRSV infection [58]. Similarly, pigs resistant to classic swine fever virus (CSFV) or foot and mouth disease virus were produced through knock-in of the corresponding shRNA, respectively [59,60]. Overexpression of the porcine *RSAD2* gene conferred protection against both PRV and CSFV infections in pigs [61]. Examples also include the insertion of an extra copy of the porcine *CD28* gene into the genome, which improved the protective immune responses to PRRSV infection in pigs [62]. In addition to the described antibacterial activities of PBD-2, previous studies have demonstrated that PBD-2 could inhibit the proliferation of two major swine viruses, PRRSV and PRV, in different cell models [6,17]. These prompt us to investigate whether PBD-2 TG pigs can resist swine viral pathogen infections in the future. Since the antimicrobial ability of PBD-2 and protein expression levels are respectively and positively correlated with their concentrations and gene copy number [6,63], a high-copy number of the gene coding for PBD-2 would further improve the disease-resistant abilities of animals. In this case, new TG pigs carrying dual *pbd-2* genes at the porcine *Rosa26* locus were generated at our lab recently [19], which should exhibit greater disease-resistant traits than the TG pigs used in this study.

In summary, as adjudged through milder clinical manifestations, less marked pathological changes, and smaller numbers of bacteria recovered from lung and brain tissues, the TG pigs overexpressing PBD-2 were more resistant to *G. parasuis* infection in comparison to their WT littermates. In addition, lung and brain homogenates from the TG pigs conferred greater bactericidal activities against *G. parasuis* than those from the WT pigs. The findings of this study suggest a promising prospect for the application of PBD-2 TG pigs in breeding disease-resistant animals and provide a feasible method to combat *G. parasuis* infection.

## 4. Materials and Methods

### 4.1. Peptides, Bacterial Strains, and Animals

Mature PBD-2 (UniProtKB accession number: Q6R953; a 37-amino acid peptide: DHYICAKKGGTCNFSPCPLFNRIEGTCYSGKAKCCIR) with a molecular mass of 4085.82 was synthesized by NewEast Biosciences (Wuhan, China). Nighty-eight percent pure synthetic PBD-2 was achieved by reversed-phase high-performance liquid chromatography and verified by electrospray ionization mass spectrometry. The synthetic PBD-2 was dissolved in Dulbecco’s phosphate-buffered saline (DPBS; Thermo Fisher Scientific, Waltham, MA, USA) for further use.

The *G. parasuis* clinical isolate SH0165 (serovar 5) and other 14 reference strains of different serovars were either cultured on tryptic soy agar (TSA; BD, Franklin Lakes, NJ, USA) or in brain heart infusion broth (BHI; BD) supplemented with 5% newborn calf serum (NBCS; TIANHANG, Huzhou, China) and 10 µg/mL nicotinamide adenine dinucleotide (NAD; Sigma-Aldrich, St. Louis, MO, USA) at 37 °C with 5% CO_2_ [64]. 

The third-generation offspring of the TG pigs overexpressing PBD-2, generated by random integration of an extra copy of the gene coding for PBD-2 in our previous study, were genotyped by PCR using a specific primer pair (NP03: 5′-GCTGGTTGTTGTGCTGTCTC-3′ and NP04: 5′-AGGTCCCTTCAATCCTGTTG-3′), as described elsewhere [15]. Animal experiments were conducted strictly in accordance with the Hubei Regulations for the Administration of Affairs Concerning Experimental Animals and were approved by the Scientific Ethical Committee for Experimental Animals of Huazhong Agricultural University (Ethics code: HZAUSW-2018-017).

### 4.2. Determination of Antibacterial Activity of PBD-2

The bacterial culture of different *G. parasuis* serovars, including *G. parasuis* strain SH0165 (serovar 5), was ten-fold diluted to a bacterial density of 5 × 10^5^ colony-forming units (CFU)/mL in DPBS. The synthetic mature PBD-2 peptide was dissolved and diluted in DPBS. A ten-microliter droplet of the diluted bacterial culture was then mixed with 90 μL of either tissue homogenates or PBD-2 solutions at different final concentrations (25, 50, 100, 150, and 200 μg/mL). The mixture was subsequently let stand for 1 h at 37 °C. After incubation, each sample was serially diluted in DPBS and then spread onto agar plates for the enumeration of surviving bacteria. Bacteria (5000 CFU) incubated with DPBS served as the control group (0 µg/mL PBD-2). 

To determine the influence of PBD-2 on the growth of bacteria, *G. parasuis* strain SH0165 was cultured overnight and then transferred to fresh medium with or without PBD-2 (500 μg/mL). Following that, the values of the optical density of bacterial cultures at 600 nm were recorded every hour for 10 h. The numbers of bacteria in each sample at the mid-log phase (4 h after cultivation) and the stationary phase (9 h after cultivation) were analyzed through bacterial colony counting.

### 4.3. Cohabitation Challenge Trial

All pigs at 28 days of age were first confirmed as antibody-negative for *G. parasuis* using the OppA ELISA kit (BioChek, Reeuwijk, Netherlands). After genotype analysis, TG pigs (*n* = 5) and their WT littermates (*n* = 6) were cohoused with 11 other WT pigs that had been intratracheally inoculated with 2 × 10^10^ CFU of *G. parasuis* SH0165, while one TG pig and two WT pigs were raised separately from the cohabitation group and used as the mock group. The rectal temperatures and clinical signs of the pigs were monitored and recorded until four days postinfection (dpi), with blind assessments implemented to score the clinical signs (feeding, breathing, and movement), according to a previous study [65]. All pigs were euthanized at 4 dpi, and pathological changes of the pigs were recorded for scoring based on the varying severity of pathological changes, including pleurisy, peritonitis, meningitis, pericarditis, hepatitis, splenitis, and pneumonia. The score for each pathological change ranged from 0 to 3, with 3 being the more severe.

### 4.4. Histopathological Analysis

Brain and lung tissues from TG and WT pigs were collected and fixed in PBS-buffered 4% formaldehyde for 48 h. Following paraffin embedding and sectioning, the sections were then subjected to HE staining. The lesions of brain and lungs were observed under a microscope and scored. The scoring criteria for lung lesions included the extent of alveolar wall thickening, inflammatory cell infiltration, and the presence of thrombi. Brain damage was scored according to the extent of bleeding or congestion, widening of perivascular space, presence of perivascular inflammatory infiltration, and meningeal edema. Each type of pathological change was assigned a score of 0–3, with 3 being the severest. Different brain and lung lesions were assessed using a subjective 0–3 score system, where 0 = none, 1 = mild, 2 = moderate, and 3 = severe.

### 4.5. Quantification of Bacterial Loads in Pig Tissues

Tissues taken from the same lung and brain regions of the WT and TG pigs were weighed and homogenized in DPBS (100 mg of each sample into 300 μL of DPBS). The homogenates were serially diluted and spread onto agar plates. The plates were left at 37 °C for 32 h, and the colonies, which were circular, pinpoint-sized, off-white, and convex, with entire edges, were counted and analyzed. *G. parasuis* colonies were randomly selected and identified by 16S rRNA PCR. The bacterial loads in tissues were calculated in CFU per gram.

### 4.6. Detection of Bactericidal Effect of Tissue Homogenates from Pigs

Lung and brain tissues at the same site from each pig were harvested. Lung or brain tissue weighing 400 mg was homogenized in 1200 μL of DPBS, followed by centrifugation at 5000× *g* for 10 min. After that, 90 μL of the supernatant was collected and heat-inactivated at 56 °C for 30 min before the subsequent bactericidal assay, as described above.

### 4.7. Statistical Analysis

Statistical analyses were conducted with GraphPad Prism 6 (GraphPad Software, La Jolla, CA, USA) using unpaired one-tailed Student’s *t*-test and showed as means ± SD. * *p* < 0.05, ** *p* < 0.01, *** *p* < 0.001, **** *p* < 0.0001.

## 5. Conclusions

In conclusion, this study identified that PBD-2 exhibited significant antibacterial abilities against *G. parasuis* in vitro and pigs overexpressing PBD-2 acquired enhanced resistance to *G. parasuis* infection. Outcomes of this study reveal the potential of breading PBD-2 TG pigs to combat infectious diseases, including Glässer’s disease, among swine herds.

## Supplementary Materials

The following are available online at https://www.mdpi.com/2079-6382/9/12/903/s1. Figure S1: Bactericidal activity of porcine β-defensin 2 (PBD-2) against different *Glaesserella parasuis* serovars. Table S1: Score for clinical signs of pigs during cohabitation. Table S2: Score for macroscopic pathological changes after dissecting pigs.
