# Supplementary material for: Pigs Overexpressing Porcine β-Defensin 2 Display Increased Resilience to Glaesserella parasuis Infection
Source: Antibiotics (Basel). 2020 Dec 14;9(12):903. doi: 10.3390/antibiotics9120903 (PMC7764891; doi:10.3390/antibiotics9120903)
Supplement: Supplementary file 1 [file antibiotics-09-00903-s001.pdf]

antibiotics

MDPI

Article

Pigs Overexpressing Porcine β-Defensin 2 Display Increased Resilience to *Glaesserella parasuis* Infection

Jing Huang [1,2,†], Xiaoyu Yang [1,2,†], Antian Wang [1,2], Chao Huang [1,2], Hao Tang [1,2], Qiuhong Zhang [1,2], Qiong Fang [1,2], Zuming Yu [1,2], Xiao Liu [1,2], Qi Huang [1,2], Rui Zhou [1,2,3,*] and Lu Li [1,2,4,*]

1 State Key Laboratory of Agricultural Microbiology, College of Veterinary Medicine, Huazhong Agricultural University, Wuhan 430070, China; jing_huang@webmail.hzau.edu.cn (J.H.); yangxiaoyu0118@webmail.hzau.edu.cn (X.Y.); wangantian@webmail.hzau.edu.cn (A.W.); huangchao_1991@webmail.hzau.edu.cn (C.H.); 2019302110155@webmail.hzau.edu.cn (H.T.); zhangqiuhong@webmail.hzau.edu.cn (Q.Z.); fangqiong@webmail.hzau.edu.cn (Q.F.); yzm@webmail.hzau.edu.cn (Z.Y.); liuxiao324@webmail.hzau.edu.cn (X.L.); qhuang@mail.hzau.edu.cn (Q.H.)

2 Cooperative Innovation Center for Sustainable Pig Production, College of Veterinary Medicine, Huazhong Agricultural University, Wuhan 430070, China

3 International Research Center for Animal Disease, Ministry of Science and Technology of China, Wuhan 430070, China

4 Key Laboratory of Development of Veterinary Diagnostic Products, Ministry of Agriculture and Rural Affairs of China, Wuhan 430070, China

* Correspondence: rzhou@mail.hzau.edu.cn (R.Z.); lilu@mail.hzau.edu.cn (L.L.)

† These authors contributed equally to this work.

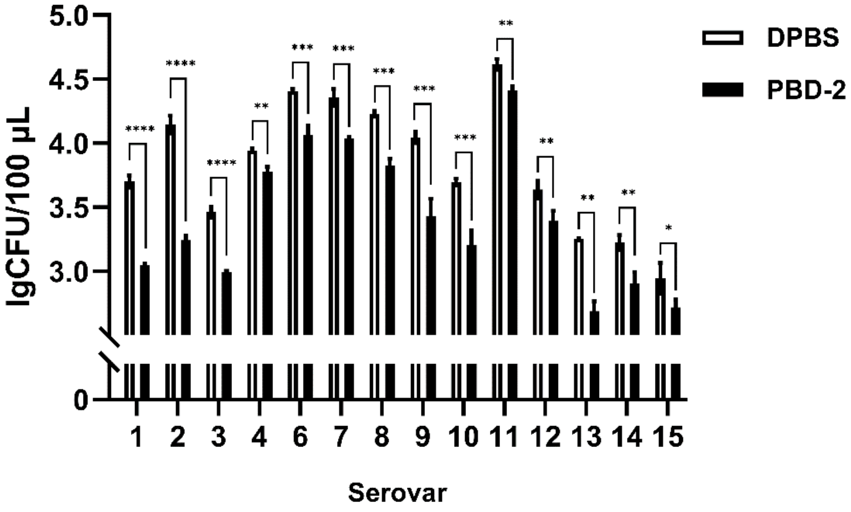

Figure S1. Bactericidal activity of porcine β-defensin 2 (PBD-2) against different *Glaesserella parasuis* serovars. PBD-2 was incubated with different *G. parasuis* serovars for 1 h before plating and counting the surviving bacteria. Bacterial culture with Dulbecco's phosphate-buffered saline (DPBS) was used as a negative control. Data are presented as mean ± SD and are plotted from three independent experiments. * $p < 0.05$, ** $p < 0.01$, *** $p < 0.001$, **** $p < 0.0001$, unpaired one-tailed Student' s *t*-test.

antibiotics

MDPI

Table S1. Score for clinical signs of pigs during cohabitation.

Number	Day 0	Day 1	Day 2	Day 3	Day 4
21 (WT)	0	2	2	1	0
23 (WT)	0	3	3	1	1
201 (WT)	0	2	2	1	0
210 (WT)	0	1	2	2	1
214 (WT)	0	3	3	2	1
217 (WT)	0	3	3	1	1
13 (TG)	0	2	2	1	0
103 (TG)	0	1	1	0	0
105 (TG)	0	3	2	1	1
202 (TG)	0	2	2	0	0
212 (TG)	0	2	2	1	1
28 (Mock)	0	0	0	0	0
86 (Mock)	0	0	0	0	0
20 (Mock)	0	0	0	0	0

Decreased appetite, shortness of breath and walking difficulty representing one point each. Wild-type (WT) and transgenic (TG) pigs were co-housed with other pigs intratracheally infected with *G. parasuis*, while pigs in the mock group were raised separately.

antibiotics

MDPI

Table S2. Score for macroscopic pathological changes after dissecting pigs.

Number	Pleurisy	Peritonitis	Meningitis	Pericarditis	Hepatitis	Splenitis	Pneumonia	Total score
21 (WT)	0	0	1	0	1	0	1	3
23 (WT)	0	0	1	0	1	0	1	3
201 (WT)	0	0	1	1	1	0	1	4
210 (WT)	0	0	1	1	1	0	1	4
214 (WT)	0	0	1	0	1	0	1	3
217 (WT)	0	0	1	0	1	0	1	3
13 (TG)	0	0	1	0	0	0	1	2
103 (TG)	0	0	1	0	0	0	0	1
105 (TG)	0	0	1	0	0	0	1	2
202 (TG)	0	0	1	0	1	0	1	3
212 (TG)	0	0	1	0	0	0	1	2
28 (Mock)	0	0	0	0	0	0	0	0
86 (Mock)	0	0	0	0	0	0	0	0
20 (Mock)	0	0	0	0	0	0	0	0

Scoring scale from 0 to 3, with 3 being the severest. WT and TG pigs were co-housed with other pigs intratracheally infected with *G. parasuis*, while pigs in the mock group were raised separately.
